# Effects of Video Game Type on Cognitive Performance and Brain Functional Connectivity: A Longitudinal EEG Study

**DOI:** 10.3390/brainsci16010024

**Published:** 2025-12-25

**Authors:** Jingqing Lu, Ruifang Cui, Lijun Jiang, Chenyu Mu, Weiyi Ma, Diankun Gong, Dezhong Yao

**Affiliations:** 1 The Clinical Hospital of Chengdu Brain Science Institute, Sichuan Institute for Brain Science and Brain-Inspired Intelligence, Tianfu Jiangxi Laboratory, University of Electronic Science and Technology of China, Chengdu 611731, China; jingqing_lu@std.uestc.edu.cn (J.L.);; 2 Center for Information in Medicine, School of Life Science and Technology, University of Electronic Science and Technology of China, Chengdu 611731, China; 3 School of Human Environmental Sciences, University of Arkansas, Fayetteville, AR 72701, USA

**Keywords:** video game genres, neuroplasticity, cognitive function, resting-state electroencephalography

## Abstract

**Background**: Previous research has shown that video gaming experience is associated with changes in cognitive and perceptual functions as well as neural structure and function. However, how the different types of video games differentially influence cognitive function and neuroplasticity remains unclear. **Methods**: In this 30-week longitudinal study, participants were randomly assigned to an action video game group or a strategy card game group. Behavioral assessments and resting-state electroencephalography (EEG) recordings were administered at six time points to evaluate changes in attention, working memory, executive function, and their neural correlates. **Results**: Both groups showed significant improvements in multiple cognitive tasks, but the underlying neural mechanisms differed. The action video game group showed greater increases in low-frequency EEG relative power (delta and theta bands) and more pronounced decreases in alpha-band functional connectivity at the 10-week follow-up after the end of training. **Conclusions**: These findings suggest that different types of video games improve cognition through distinct neuroplasticity pathways, with action games effective in optimizing neural efficiency and producing sustained effects. This study provides new insights into the cognitive and neural mechanisms of game-based enhancements and offers implications for the design of targeted digital cognitive interventions.

## 1. Introduction

Video games are an integral component of modern society. According to the Global Games Market Report, the global gaming population exceeded 3.42 billion in 2024, and the gaming industry now surpasses the film and music industries in revenue. Once regarded solely as a form of recreation, video games have evolved into a pervasive element of contemporary life, impacting diverse domains including sports [1], education [2], healthcare [3,4], and esports [5], while also offering valuable insights for cognitive neuroscience research [6,7]. Video games encompass a wide range of experiences, from slow-paced strategy games (e.g., Legends of the Three Kingdoms [TKK]) to fast-paced action games (e.g., League of Legends [LOL]). These games create rich, ecologically valid virtual environments that are both engaging and immersive, often recruiting multiple cognitive functions, including working memory, logical reasoning, attention, and rapid reaction. Such cognitively demanding gameplay provides real-world contexts in which video games can foster cognitive enhancement. Bavelier and Green have further proposed that action video games, in particular, may serve as effective training tools capable of inducing cognitive and brain plasticity [8].

Over the past few decades, a growing body of research has examined the impact of video game experience on behavior and cognition, suggesting that gaming is associated with improvements in behavior and cognitive function [9]. Among the various genres, action video games and strategic card games have emerged as two of the most popular and cognitively engaging types. Action video games (e.g., LOL) emphasize reflexes, hand-eye coordination, and rapid information processing, requiring players to monitor peripheral stimuli, track multiple targets, and make split-second decisions [10]. Prior studies have shown that action video game experts tend to outperform non-experts in executive control [11], selective visual attention [12,13], working memory [14,15], processing speed [16], and perceptual abilities [17]. A meta-analysis by Bediou et al. further confirmed the positive effects of action video games on a wide range of cognitive abilities in both cross-sectional studies and longitudinal studies [18]. Similarly, Waris et al. found that action video game experts outperformed non-experts on tasks assessing verbal working memory, visuospatial memory, and N-back working memory [15]. Additionally, cross-sectional studies have shown enhanced attentional control in action video game experts relative to non-experts [19,20], while interventional studies suggest that action video game training can broadly enhance cognition by accelerating the process of “learning to learn” [21].

In contrast, strategy card video games (e.g., TKK) emphasize working memory, strategic planning, and real-time decision-making. Players must manage card resources and analyze the battlefield situation, and coordinate character abilities and card properties to determine the optimal sequence of actions. Operating under conditions of incomplete information, they must balance short-term tactical moves with long-term strategic goals. Empirical evidence supports the cognitive benefits of such gameplay: Estrada-Plana et al. demonstrated through a double-blind randomized controlled trial that five weeks of modern card and board gameplay enhanced semantic verbal fluency and response inhibition [22]. Likewise, Basak et al. found that older adults who underwent 23.5 h of strategy video game exhibited greater improvements in task switching, working memory, and visual short-term memory compared with untrained controls [23].

Neuroscience research shows that video games can improve cognitive task performance and affect brain function and structure [24,25]. Mukherjee’s cross-sectional MRI study indicated that long-term action video gaming experience is associated with greater cortical thickness in the dorsal stream and enhanced occipito-parietal connectivity [26]. From a neuro-electrophysiology perspective, cognitive function and brain structure are closely related to resting-state EEG activity [27]. For example, Finnigan and Robertson found that higher resting theta power in healthy older adults correlates with better immediate and delayed verbal recall, attention, and executive function [28]. Similarly, Ramyarangsi et al. compared resting EEG among gymnastics, soccer, and esports athletes, reporting that esports players exhibited higher delta power and reduced alpha power at Fpz and Cz when compared to gymnasts [29]. Moreover, Klimesch proposed that alpha-band oscillations reflect fundamental cognitive processes, particularly attention suppression and selection [30].

A substantial body of research, including both cross-sectional and training intervention studies, has demonstrated that video games can enhance various cognitive functions, such as visual attention, executive control, visual short-term memory, and processing speed. For example, Dobrowolski et al. compared participants playing first-person shooter and real-time strategy games, suggesting that the cognitive benefits of video gaming may vary by genre [31]. However, a critical gap remains: no longitudinal intervention studies have directly compared different types of video game training in terms of their effects on cognitive function and associated changes in brain structure and function. Specifically, there is limited evidence regarding whether different game genres exert distinct, type-dependent effects on cognition and neural activity. To address this gap, the present study conducted a 30-week longitudinal gaming experiment, collecting behavioral and resting-state EEG data at multiple time points for feature analysis and evaluation. This study systematically compared the effects of two types of game training—action video games and strategy card games—on spatial attention, spatial working memory, executive function, and EEG measures. We adopted three classic, domain-specific psychological paradigms (Distributed Spatial Attention task, Spatial N-back task, and Color-Direction Conflict task) to operationalize and measure the three focal cognitive domains. Each paradigm, through its structured design and objective performance indicators, directly indexes a distinct core cognitive domain, as corroborated by extensive cognitive psychology and neuroscience. The Distributed Spatial Attention paradigm was adapted from the spatial vigilance and multi-location monitoring protocols pioneered by Posner [32,33], who first demonstrated that spatial attention can be selectively oriented and distributed across non-contiguous regions independent of eye movements. The N-back paradigm is the gold-standard assay for visuospatial working memory [34], as defined by Jonides et al.’s seminal working memory model [35], which outlines the core functions of temporary information maintenance and online manipulation. The Color-Direction Conflict paradigm is a variant of the classic Stroop paradigm [36]. This paradigm measures executive function by requiring participants to respond to one stimulus attribute while inhibiting prepotent responses to a conflicting attribute [37]. We hypothesized that both games improve cognitive performance and induce changes in EEG features, with action game training producing greater cognitive gains and neural modulation due to its higher cognitive demands.

In summary, this study addresses a significant gap in the research on the differential effects of long-term training with different types of video games. It aims to provide new experimental evidence elucidating the mechanisms by which gaming induces neuroplasticity. By integrating behavioral and EEG data, this study will illuminate the dynamic neural processes underlying cognitive task performance and resting-state EEG activity during long-term gaming training. This study offers new insights for designing personalized and targeted cognitive intervention programs, as well as for the development of functionally enhancing games. Moreover, this study further supports the use of resting-state EEG features as objective markers for evaluating the effectiveness of cognitive interventions.

## 2. Materials and Methods

### 2.1. Participants

Ninety participants (mean age = 19.83 (±1.31) years; range: 18–22 years; 74 males), who were students at the Chengdu University of Information Technology, were recruited. The recruitment process was implemented through a multi-channel, multi-stage procedure. First, printed and electronic recruitment posters with clear descriptions of the study’s purpose, basic procedures, time commitment, and financial compensation were released via the student group WeChat groups and bulletin boards in teaching buildings and dormitory complexes. Second, interested students completed a preliminary screening questionnaire that collected information, such as age, gender, contact information, video game experience, psychological scales, and physical health status. Third, eligible participants were contacted for a brief offline interview to confirm their eligibility. During this interview, researchers clarified the risks of the study and ensured that the participants fully understood the research protocol. Finally, all enrolled participants signed a written informed consent form before participating in the formal experiment and were informed of their right to withdraw from the study at any time without penalty. Participants were compensated upon completion of the study. This study was approved by the Research Ethics Committee of the University of Electronic Science and Technology of China (UESTC), and all procedures adhered to the Helsinki Declaration.

Participants were randomly assigned to either the LOL group, a typical action video game, or the TKK group, a typical strategy card game. It is noteworthy that all participants in this study had no long-term video game experience, defined as less than fifty hours of cumulative regular gaming experience over the past year, and no regular gaming behavior with a frequency exceeding once per month. All participants were right-handed, in good health, maintained consistent daily routines, had normal or near-normal vision, and had no history of neurological disease, internet addiction, or substance abuse. After excluding participants who withdrew or whose data quality was insufficient, the final sample included 33 participants (mean age = 19.91 ± 1.31 years; 27 males) in the LOL group and 35 participants (mean age = 19.80 ± 1.29 years; 28 males) in the TKK group.

### 2.2. Experimental Design

This study employed a between-subjects design, with participants randomly assigned to either the LOL group or the TKK group. We selected LOL (a multiplayer online battle arena, categorized as an action-oriented competitive game) mainly because LOL is a mainstream experimental paradigm in the fields of cognitive psychology and game behavior research. Previous studies had used it as a typical representative of real-time competitive games to explore its cognitive mechanisms, ensuring the comparability and relevance of the results of this study with previous findings. We selected TKK (a turn-based strategy card game) mainly because it is a typical example of a localized strategy game and has a clear strategic decision-making chain and a clear cognitive requirement dimension. Moreover, it can form a high-quality “action-strategy” cognitive comparison paradigm with LOL. The two games differ drastically in cognitive demand profiles, which allows us to isolate and compare the specific cognitive mechanisms underlying different types of gaming experiences. LOL requires real-time reactive cognition, including rapid visual attention allocation (tracking multiple in-game targets simultaneously), fine motor coordination (executing precise skill releases), dynamic team collaboration (real-time communication and role adjustment), and immediate decision-making under time pressure (responding to enemy attacks or ally requests within 1–2 s). By contrast, TKK relies on deliberative strategic cognition, such as logical deduction, probabilistic reasoning, long-term strategy planning, and information asymmetry-based game theory. Additionally, League of Legends imposes a higher working memory load for real-time state monitoring, while TKK emphasizes episodic memory for tracking historical card plays and opponent behavioral patterns.

The study lasted approximately seven months, including five months of game training, during which participants completed one hour of gameplay per day, five days per week. Before the initiation of the experiment, all participants were clearly informed of the behavioral guidelines: they could only participate in the target game training designated for this study and should avoid engaging in any non-target game activities as much as possible. During the experiment, self-reported game activity follow-ups were conducted weekly to monitor participants’ gaming behavior. Additionally, with the voluntary and informed authorization of participants, official gameplay data were retrieved from their game accounts to perform cross-validation of their self-reported gaming behaviors. This ensured the accuracy and credibility of the behavioral monitoring data.

**Figure 1:**
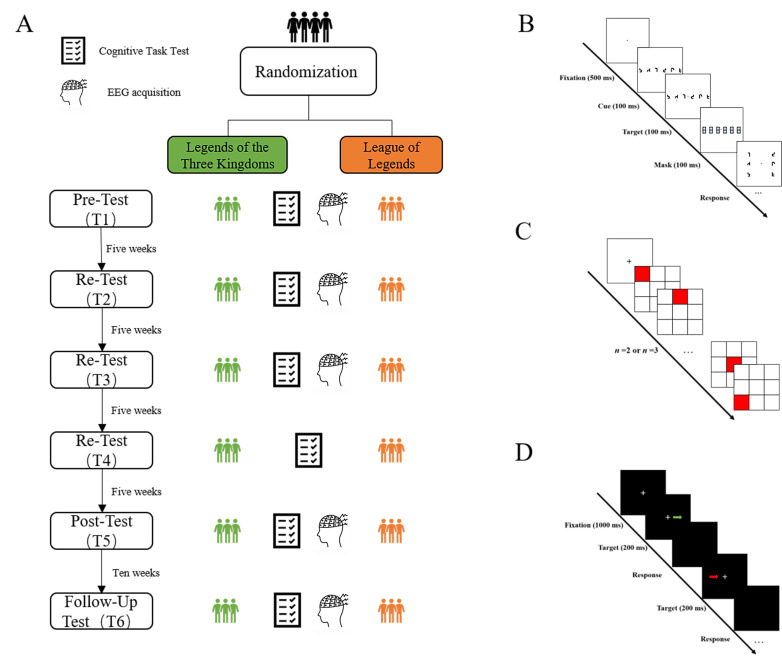
Overall experimental paradigm. (**A**) Detailed experimental process. (**B**) Detailed process of the Distributed Spatial Attention task. (**C**) Detailed process of the Spatial N-back task. (**D**) Detailed process of the Color-Direction Conflict task.

Assessments were conducted at six time points, including five eyes-closed resting-state EEG recordings (T1, T2, T3, T5, T6) and six cognitive-behavioral task sessions (T1–T6). T1 served as the baseline pretest; T2 was administered after five weeks of training; T3 after 10 weeks; and T4 after 15 weeks, including only the cognitive-behavioral tasks without EEG recording. T5 was the posttest, administered 20 weeks after the onset of the training. T6 was the retention test, administered 10 weeks after the conclusion of the training. We set the training period as 30 weeks based on two rationales. First, previous studies on cognitive training have demonstrated that short-term interventions (≤12 weeks) often only induce transient cognitive improvements, whereas longer-term training (≥24 weeks) is required to produce stable, lasting changes in cognitive performance and underlying neural mechanisms [38,39]. Second, considering the complexity of the strategy card games used in our study, participants need enough time to master game rules and develop strategic thinking patterns—a process that typically takes 4–6 weeks of regular practice. The cognitive-behavioral task comprised three tasks: the Distributed Spatial Attention task, assessing distributed spatial attention; the Spatial N-back task, evaluating spatial working memory; and the Color-Direction Conflict task, measuring executive function. Each task included an instructional overview and practice trials before the formal assessment. The overall experimental flow is illustrated in Figure 1.

In the Distributed Spatial Attention task, each trial began with a central fixation point, followed by the rapid presentation of three meaningless characters on either side. A red dot then briefly appeared below one of the characters, and participants were instructed to remember the shape of the character corresponding to the red dot’s position. A masking display with three rectangular patterns subsequently appeared, followed by a probe display containing a red central fixation point and three characters on each side. Participants were asked to identify and select, using the left mouse button, the character previously associated with the red dot.

The Spatial N-back task included two difficulty levels (*n* = 2 or 3). Each trial began with a central fixation cross, followed by the presentation of a 3 × 3 grid in which red squares were sequentially illuminated. Participants were instructed to indicate whether the current red square appeared in the same position as the square presented “n” trials earlier (*n* = 2 or 3) by pressing the corresponding letter key. No responses were required for the first n trials.

In the Color-Direction Conflict task, each trial began with a fixation cross, followed by the presentation of a colored arrow on either side of the fixation point. Participants were instructed to respond as quickly as possible according to the color–direction rule: a green arrow pointing right required pressing the ‘P’ key with the right hand; a green arrow pointing left required pressing the ‘Q’ key with the left hand; a red arrow pointing to the right required pressing the ‘Q’ key with the left hand; a red arrow pointing to the left required pressing the ‘P’ key with the right hand.

### 2.3. Data Acquisition, Preprocessing, and Analysis

EEG recording and cognitive-behavioral testing were conducted in separate sessions. Participants were instructed to maintain regular sleep, refrain from smoking, and avoid caffeine consumption for one week prior to data collection. All sessions were conducted in a quiet, temperature-controlled laboratory free from external distractions. Cognitive-behavioral tasks were administered via a computer-based interface, with brief breaks permitted between tasks. To minimize potential order effects, the sequence of the three cognitive tasks was randomized for each participant.

EEG data were recorded using a 32-channel amplifier (Brain Products GmbH, Gilching, Germany), with electrodes positioned according to the international 10–20 system. The EEG sampling rate was set to 1000 Hz, electrode impedance was maintained below 5 kΩ, and all electrodes were referenced to the Fz electrode. Before recording, participants were instructed to sit comfortably, regulate their breathing, and minimize movement. They were then asked to close their eyes and rest their chins on the headrest to minimize head motion artifact. Five minutes of eyes-closed resting-state EEG data were subsequently recorded.

Behavioral data from the three cognitive tasks were organized using Microsoft Excel. Resting-state EEG data were preprocessed offline in MATLAB 2022b using custom scripts based on EEGLAB. The preprocessing steps included restoration of the original Fz signal, band-pass filtering (1–40 Hz) and notch filtering (50 Hz), artifact correction using EOG regression and residual artifact removal, interpolation of bad channels, and re-referencing to the REST reference [40]. The final dataset consisted of clean EEG signals referenced to the REST reference.

EEG frequency bands were defined as follows: delta (1–4 Hz), theta (4–8 Hz), alpha (8–12 Hz), beta (12–30 Hz), gamma (30–40 Hz), and the full band (1–40 Hz). The preprocessed EEG data were segmented into 5 s epochs. Fast Fourier Transform (FFT) was applied to each epoch to calculate spectral power indices within the delta, theta, alpha, beta, and gamma bands. Resting-state power indices were then averaged across all epochs. The absolute power value is calculated by

(1) $$Y_{power}=10\times log10\left(\frac{{\left\|\frac{2}{0.375}\times Y\right\|}^{2}}{window\;size}\right)$$

where Y is a complex number calculated by FFT, $\left\|.\right\|$ is a complex modulus operation (using ‘abs’ function of MATLAB), the unit is $10\cdot log10{\mu V}^{2}/Hz$, andthe window size is calculated by max(pow2(nextpow2(length(epoch))−3),4). Pow2 and nextpow2 are functions incorporated in MATLAB that are specifically designed for the computation of powers of 2. Noting that, multiplying by 2 accounts for negative frequencies, and counteracts the reduction by a factor of 0.375 that occurs as a result of cosine (Hann) tapering. $Relative\;power\;in\;a\;specific\;band=\frac{power\;of\;\;specific\;band}{total\;power\;across\;full\;band}.$ The relative power of each frequency band was averaged across all scalp electrodes to generate a global index of oscillatory activity for subsequent statistical analyses.

Additionally, Phase-Locking Values (PLV) were computed from the preprocessed EEG epochs to evaluate network connectivity across electrodes within each frequency band [41]. Considering the influence of volume conduction, neighboring scalp electrodes often capture overlapping cortical activity and exhibit similar signal patterns [42]. To mitigate this effect in subsequent analyses, twenty electrodes (Fp1, Fp2, F7, F3, Fz, F4, F8, T7, C3, Cz, C4, T8, P7, P3, Pz, P4, P8, O1, O2, and Oz) were selected for constructing the resting-state functional network. For two EEG signals with data length L, the PLV is defined as

(2) $$PLV=\left|\frac{1}{L}\sum_{t=0}^{L}e^{i\emptyset \left(t\right)}\right|$$

where L is the EEG data length, t denotes the time index covering all time segments of the EEG data (with a value range from 0 to L−1), and $\emptyset \left(t\right)$ represents the instantaneous phase difference between the two EEG signals at the t-th sampling point. The phase locking index is sensitive to phase change, and its value ranges from 0 to 1. The PLV=1 if and only if the condition of strict phase locking is obeyed. In contrast, the PLV=0 for uniformly distributed phases.

All statistical analyses were conducted using MATLAB 2022b (The MathWorks, Natick, MA, USA) and GraphPad Prism 9.0 software (GraphPad Software, San Diego, CA, USA). For within-group comparisons of training effects, data were first examined using Mauchly’s test of sphericity and normality tests. When data did not conform to a normal distribution, nonparametric tests (Friedman tests) followed by Dunn’s multiple comparisons test were applied. When data met the assumption of normality, a one-way repeated-measures Analysis of Variance (ANOVA) was conducted; if the sphericity assumption was violated, the Geisser-Greenhouse correction was applied. Tukey’s multiple comparisons test was used for post hoc analysis. For between-group comparisons of training effects, a two-way repeated-measures ANOVA was employed, with a 2 × 6 (group × time) design for behavioral data and a 2 × 5 (group × time) design for EEG data. When a significant main effect of group was observed, simple effects analyses were conducted, and Bonferroni’s test or Tukey’s multiple comparisons test was used to control for the Type I error in subsequent pairwise comparisons. To explore the relationship between the behavioral and EEG data, we further conducted a correlation analysis between EEG indicators (relative power of the delta and theta bands and brain functional network connectivity) and behavioral task performance. Given the dynamic fluctuations in the physiological and cognitive states of participants during the training period, to control for the interference of state heterogeneity on the association results, this analysis only included paired data from the baseline period (pre-test) and the last test (follow-up test).

## 3. Results

### 3.1. Behavioral Results

The behavioral task results showed that both types of game training significantly improved participants’ distributed spatial attention, spatial working memory, and executive function. Moreover, the LOL group demonstrated greater improvement and longer-lasting effects on spatial attention and spatial working memory than the TKK group.

**Figure 2:**
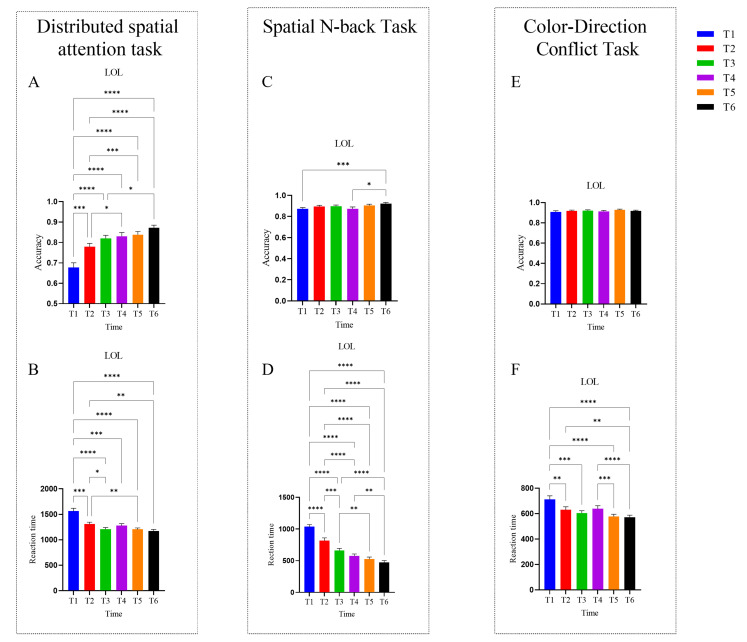
Intra-group behavioral results for the LOL group. (**A**) Accuracy in the Distributed Spatial Attention task. (**B**) Reaction time in the Distributed Spatial Attention task. (**C**) Accuracy in the Spatial N-back task. (**D**) Reaction time in the Spatial N-back task. (**E**) Accuracy in the Color-Direction Conflict task. (**F**) Reaction time in the Color-Direction Conflict task. Error bars represent the standard error of the mean. * *p* < 0.05, ** *p* < 0.01, *** *p* < 0.001, **** *p* < 0.0001.

The intra-group behavioral results for the LOL group are shown in Figure 2. For the Distributed Spatial Attention task, both accuracy (*F* (3.474,111.2) = 26.77, *p* < 0.0001, *partial η*^2^ = 0.456) and reaction time (*F* (2.621,83.87) = 24.73, *p* < 0.0001, *partial η*^2^ = 0.436) exhibited a significant main effect of time, indicating substantial improvements in distributed spatial attention over the course of training. Tukey’s post hoc test results revealed significant accuracy improvements across most time points, except for T3 vs. T2, T3 vs. T4, T3 vs. T5, T4 vs. T5, T4 vs. T6, and T5 vs. T6. Significant improvements in accuracy were observed across all other time-point comparisons (*p’s* < 0.05). Reaction time significantly decreased between T1 and all subsequent times, as well as between T2 and T3, T2 and T5, and T2 and T6 (*p’s* < 0.05).

For the Spatial N-back task, accuracy (*Friedman statistic* = 19.86, *p* < 0.01, *df* = 5) and reaction time (*F* (3.597,115.1) = 77.66, *p* < 0.0001, *partial η*^2^ = 0.708) both exhibited significant main effects of time, indicating significant improvements in spatial working memory. Dunn’s post hoc test results indicated significant accuracy gains for the comparisons T1 vs. T6, and T4 vs. T6 (*p’s* < 0.05). Tukey’s post hoc test results showed that reaction time decreased significantly across nearly all time points (*p’s* < 0.05) except T3 vs. T4, T4 vs. T5, and T5 vs. T6.

For the Color-Direction Conflict task, accuracy (*F* (3.898,124.7) = 1.328, *p* = 0.264, *partial η*^2^ = 0.04) did not show a significant main effect of time, whereas reaction time (*Friedman statistic* = 69.32, *p* < 0.0001, *df* = 5) did. Dunn’s post hoc test results revealed significant reaction time reductions for the comparisons T1 vs. T2, T1 vs. T3, T1 vs. T5, T1 vs. T6, T2 vs. T6, T4 vs. T5, and T4 vs. T6 (*p’s* < 0.05).

**Figure 3:**
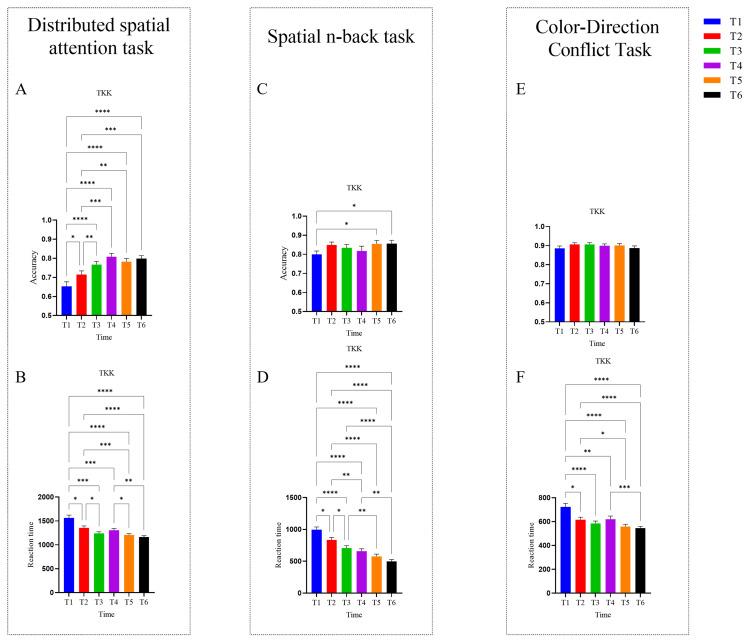
Intra-group behavioral results data for the TKK group. (**A**) Accuracy in the Distributed Spatial Attention task. (**B**) Reaction time in the Distributed Spatial Attention task. (**C**) Accuracy in the Spatial N-back task. (**D**) Reaction time in the Spatial N-back task. (**E**) Accuracy in the Color-Direction Conflict task. (**F**) Reaction time in the Color-Direction Conflict task. Error bars represent the standard error of the mean. * *p* < 0.05, ** *p* < 0.01, *** *p* < 0.001, **** *p* < 0.0001.

The intra-group behavioral results for the TKK group are shown in Figure 3. For the Distributed Spatial Attention task, both accuracy (*F* (4.445, 151.1) = 21.32, *p* < 0.0001, *partial η^2^* = 0.386) and reaction time (*F* (2.65, 90.8) = 21.09, *p* < 0.0001, *partial η^2^* = 0.383) exhibited a significant main effect of time, indicating substantial improvements in distributed spatial attention over the course of training. Tukey’s post hoc test results revealed significant accuracy improvements for the comparisons T1 vs. T2–T6, and T2 vs. T3–T6 (*p’s* < 0.05). Reaction time decreased significantly between most time points, except T2 vs. T4, T3 vs. T4, T3 vs. T5, T3 vs. T6, and T5 vs. T6. Significant improvements in reaction time were observed across all other time-point comparisons (*p’s* < 0.05).

For the Spatial N-back task, accuracy (*Friedman statistic* = 13.46, *p* < 0.05, *df* = 5) and reaction time (*F* (3.933,133.7) = 44.23, *p* < 0.0001, *partial η^2^* = 0.565) both exhibited significant main effects of time, indicating improved spatial working memory. Dunn’s post hoc test results indicated significant accuracy gains for the comparisons T1 vs. T5, and T1 vs. T6. Reaction time decreased significantly across most time points, except T3 vs. T4, T4 vs. T5, and T5 vs. T6. Significant improvements in reaction time were observed across all other time-point comparisons (*p’s* < 0.05).

For the Color–Direction Conflict task, accuracy (*Friedman statistic* = 4.852, *p* = 0.4342, *df* = 5) did not exhibit a significant main effect of time, whereas reaction time did (*Friedman statistic* = 81.53, *p* < 0.0001, *df* = 5). Dunn’s post hoc test results revealed significant reductions in reaction time for the comparisons T1 vs. T2–T6, T2 vs. T5–T6, and T4 vs. T6 (*p’s* < 0.05).

**Figure 4:**
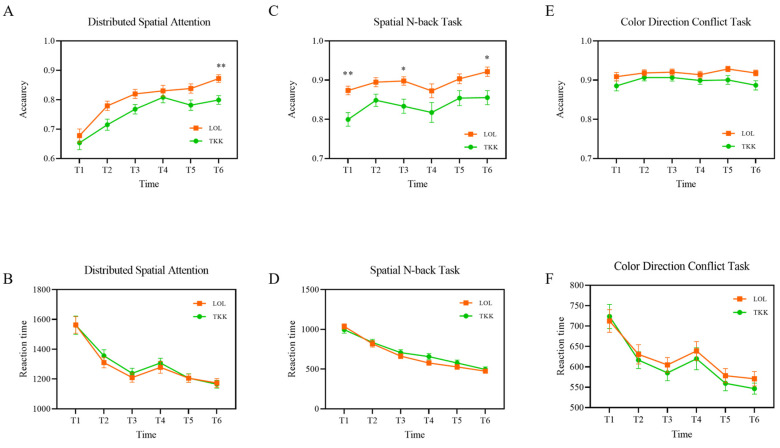
Inter-group behavioral results between the LOL and TKK groups. (**A**) Accuracy in the Distributed Spatial Attention task. (**B**) Reaction time in the Distributed Spatial Attention task. (**C**) Accuracy in the Spatial N-back task. (**D**) Reaction time in the Spatial N-back task. (**E**) Accuracy in the Color-Direction Conflict task. (**F**) Reaction time in the Color-Direction Conflict task. Error bars represent the standard error of the mean. * *p* < 0.05, ** *p* < 0.01.

Figure 4 shows the results of the two-way repeated-measures ANOVA for behavioral data from the TKK and LOL groups. For the Distributed Spatial Attention task, the main effect of group was significant (*F* (1, 66) = 6.74, *p* < 0.05, *partial η*^2^ = 0.243), with no significant time × group interaction (*F* (5, 330) = 1.293, *p* = 0.266). Post hoc Bonferroni-corrected comparisons indicated that the LOL group showed significantly higher accuracy than the TKK group at T6 (*p* < 0.01; Figure 4A).

For the Spatial N-back task, the main effect of group was also significant (*F* (1, 66) = 12.56, *p* < 0.001, *partial η*^2^ = 0.358), with no significant time × group interaction (*F* (5, 330) = 0.361, *p* = 0.874). Post hoc Bonferroni-corrected comparisons revealed that the LOL group had significantly higher accuracy than the TKK group at T1, T3, and T6 (*p’s* < 0.05; Figure 4C). We noted baseline heterogeneity in the accuracy of Spatial N-back (T1). Then, we conducted a repeated-measures analysis of covariance (ANCOVA) equivalent with the baseline accuracy of the Spatial N-back task (T1) as the covariate. The supplementary ANCOVA results demonstrated that there were no significant differences in accuracy between groups.

For the Color-Direction Conflict task, no significant differences in accuracy were observed between groups (Figure 4E). Likewise, reaction times for all three cognitive tasks did not show significant main effects or interactions (Figure 4B,D,F).

### 3.2. EEG Results

Analysis of resting-state EEG showed that both types of game training increased the relative power of low-frequency bands (delta and theta) and decreased alpha-band functional connectivity. The LOL group exhibited greater and more sustained enhancements in low-frequency power and stronger reductions in alpha-band connectivity compared with the TKK group. Additionally, we conducted repeated-measures ANOVA for the relative power of the EEG in other frequency bands, including alpha, beta, and gamma bands. The results revealed no significant main effects of group or group × time interaction effects for these frequency bands, indicating no statistically significant substantial changes in these ranges.

**Figure 5:**
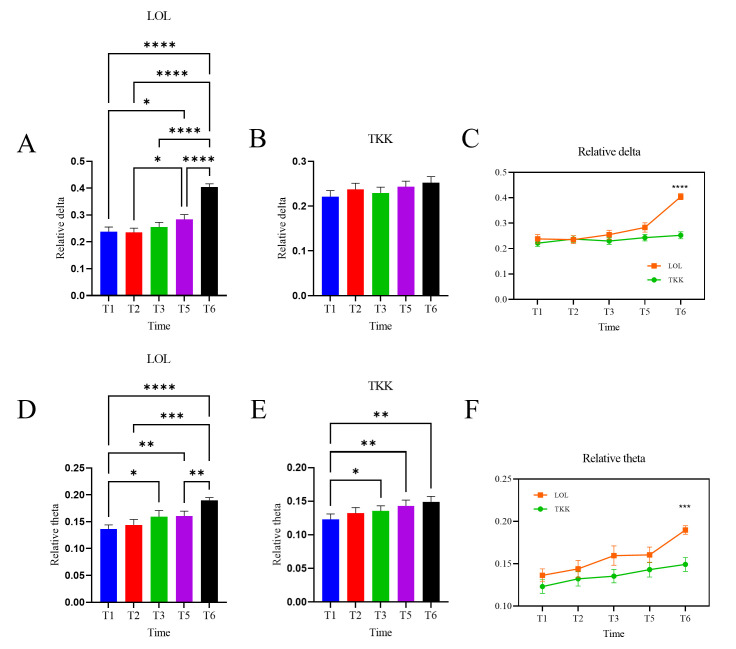
Intra-group and inter-group results of the EEG power of the LOL and TKK groups. (**A**) Relative delta band power in the LOL group. (**B**) Relative delta band power in the TKK group. (**C**) Relative delta band power. (**D**) Intra-group results for relative theta band power in the LOL group. (**E**) Intra-group results for relative theta band power in the TKK group. (**F**) Inter-group results for relative theta band power. Error bars represent the standard error of the mean. * *p* < 0.05, ** *p* < 0.01, *** *p* < 0.001, **** *p* < 0.0001.

Figure 5 shows the intra-group and inter-group results for relative delta and theta band power in resting-state EEG for the LOL and TKK groups. In the LOL group, both relative delta band power (*F* (2.326,74.45) = 40.77, *p* < 0.0001, *partial η*^2^ = 0.560) and relative theta band power (*F* (2.725,87.19) = 13.22, *p* < 0.0001, *partial η*^2^ = 0.292) exhibited significant main effects of time, indicating progressive increases in low-frequency EEG power throughout training (Figure 5A,D). Tukey’s post hoc tests revealed significant improvements in relative delta power across most time points (*p’s* < 0.05) except T1–T2, T1–T3, T2–T3, and T3–T5, and in relative theta power except T1–T2, T2–T3, T3–T5, T3–T6, and T2–T5.

In the TKK group, relative theta power showed a significant time effect (*F* (2.680, 91.12) = 5.94, *p* < 0.01, *partial η*^2^ = 0.139), with significant increases from T1 to T3, T5, and T6 (*p’s* < 0.05; Figure 5E). Although relative delta power in the TKK group did not reach significance (*F* (2.818, 95.82) = 1.96, *p* = 0.13, *partial η*^2^ = 0.054), it showed an increasing trend over time (Figure 5B).

For the relative delta power, the main effect of group was significant (*F* (1, 66) = 7.705, *p* < 0.01, *partial η*^2^ = 0.175), with significant time × group interaction (*F* (4, 264) = 19.43, *p* < 0.001, *partial η*^2^ = 0.227). Post hoc Bonferroni-corrected multiple comparisons revealed that the LOL group had significantly higher relative delta than the TKK group at T6 (*p* < 0.0001; Figure 5C). For the relative theta power, the main effect of group was significant (*F* (1, 66) = 4.24, *p* < 0.05, *partial η*^2^ = 0.154), with significant time × group interaction (*F* (4, 264) = 2.874, *p* < 0.05, *partial η*^2^ = 0.041). Post hoc Bonferroni-corrected multiple comparisons revealed that the LOL group had significantly higher relative theta than the TKK group at T6 (*p* < 0.001; Figure 5F).

**Figure 6:**
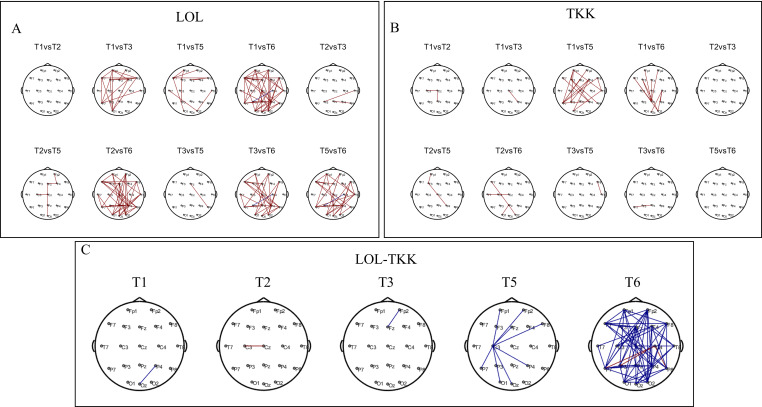
Intra-group and inter-group results of the alpha-band network connectivity in the LOL and TKK groups. (**A**) Intra-group results for the alpha-band network connectivity in the LOL group. (**B**) Intra-group results for the alpha-band network connectivity in the TKK group. (**C**) Inter-group results for the alpha-band network connectivity. Red lines indicate increased connectivity, and blue lines indicate decreased connectivity.

Figure 6 shows the intra-group and inter-group results for alpha-band brain network connectivity in resting-state EEG for the LOL and TKK groups. In the LOL group, alpha-band network connectivity showed a significant main effect of time (*p* < 0.05), indicating a progressive reduction in connectivity over the course of training (Figure 6A). Tukey’s post hoc tests revealed significant decreases for T1–T3, T1–T5, T1–T6, T2–T6, T3–T6, and T5–T6 (*p’s* < 0.05). Similarly, in the TKK group, alpha-band connectivity also exhibited a significant main time effect (*p* < 0.05), with significant reductions for T1–T5 and T1–T6 (Figure 6B). Inter-group comparisons showed that at T5 and T6, alpha-band network connectivity in the LOL group was significantly lower than that in the TKK group (*p’s* < 0.05; Figure 6C).

### 3.3. Correlation Analysis Results

Table 1 shows the correlation analysis results between behavioral cognitive task indicators and the EEG relative power of delta band. Delta band relative power was significantly positively correlated with the accuracy of the Distributed Spatial Attention task (*r* = 0.3677, *p* < 0.0001). Delta band relative power was significantly negatively correlated with the reaction time of the Distributed Spatial Attention task (*r* = −0.3341, *p* < 0.0001). Delta band relative power was significantly positively correlated with the accuracy of the Spatial N-back task (*r* = 0.3326, *p* < 0.0001). Delta band relative power was significantly negatively correlated with the reaction time of the Spatial N-back task (*r* = −0.3160, *p* < 0.001). However, the relative power of the delta band was not significantly correlated with the accuracy and reaction time of the Color-Direction Conflict task.

Table 2 shows the correlation analysis results between behavioral cognitive task indicators and the EEG relative power of the theta band. Theta band relative power was significantly positively correlated with the accuracy of the Distributed Spatial Attention task (*r* = 0.3284, *p* < 0.0001). Theta band relative power was significantly negatively correlated with the reaction time of the Distributed Spatial Attention task (*r* = −0.2453, *p* < 0.001). Theta band relative power was significantly positively correlated with the accuracy of the Spatial N-back task (*r* = 0.1999, *p* < 0.05). Theta band relative power was significantly negatively correlated with the reaction time of the Spatial N-back task (*r* = −0.2788, *p* < 0.01). However, the relative power of the theta band was not significantly correlated with the accuracy and reaction time of the Color-Direction Conflict task.

**Figure 7:**
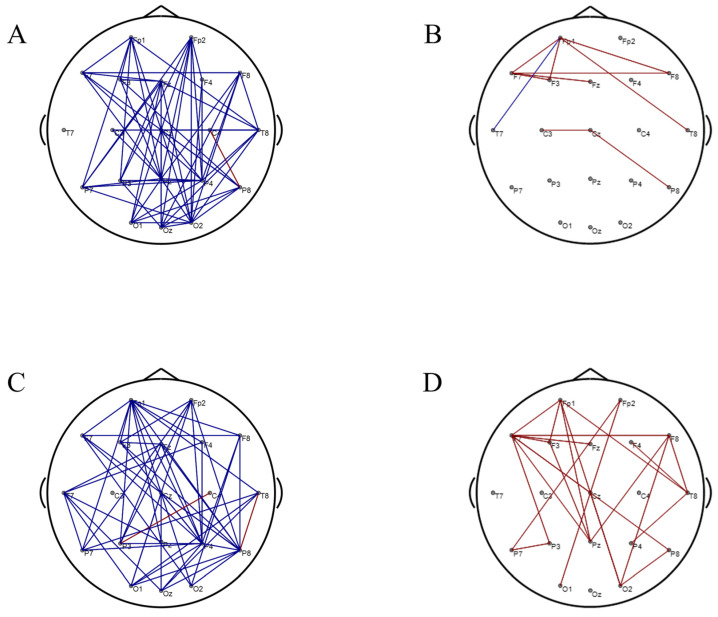
Correlation analysis results between behavioral cognitive task indicators and alpha band brain network connectivity. (**A**) Correlation analysis results between the accuracy of the Distributed Spatial Attention task and alpha band brain network connectivity. (**B**) Correlation analysis results between the reaction time of the Distributed Spatial Attention task and alpha band brain network connectivity. (**C**) Correlation analysis results between the accuracy of the Spatial N-back task and alpha band brain network connectivity. (**D**) Correlation analysis results between the reaction time of the Spatial N-back task and alpha band brain network connectivity. Red lines represent a positive correlation, and blue lines represent a negative correlation.

Figure 7 shows the correlation analysis results between behavioral cognitive task indicators and alpha band brain network connectivity. Correlation analysis results showed that the connectivity between the frontoparietal and occipital lobes was significantly negatively correlated with the accuracy of the Distributed Spatial Attention and the Spatial N-back tasks (Figure 7A,C). Additionally, the connectivity between the frontoparietal and occipital lobes was significantly positively correlated with the reaction time of the Distributed Spatial Attention and the Spatial N-back tasks (Figure 7B,D). However, alpha band brain network connectivity was not significantly correlated with the accuracy and reaction time of the Color-Direction Conflict task.

## 4. Discussion

This 30-week longitudinal study compared the effects of the action video game (LOL) and the strategy card game (TKK) training on cognitive function and resting-state EEG activity. Both training regimens significantly enhanced performance across three cognitive tasks: the Distributed Spatial Attention task, the Spatial N-back task, and the Color-Direction Conflict task. Neurophysiological analyses revealed that both groups exhibited increased relative delta and theta EEG power alongside decreased alpha-band brain network connectivity. Between-group comparisons indicated that LOL training produced stronger neuroplastic effects: at T6, the LOL group outperformed the TKK group in the Distributed Spatial Attention, accompanied by pronounced increases in low-frequency EEG relative power and a corresponding decrease in alpha-band functional connectivity.

With respect to cognitive-behavioral tasks, this study demonstrates that video game training can substantially enhance cognitive performance, consistent with prior research findings [9,13,43]. While repeated cognitive testing (administered once every five weeks) may have introduced mild practice effects that contributed to baseline-to-post-test performance increments, two design features mitigated this confounding factor: (1) The 5-week assessment interval was long enough to attenuate cumulative skill gains from test exposure, and (2) the randomized administration order of cognitive tasks prevented the formation of fixed response habits. Most critically, practice effects were uniformly distributed across all experimental groups, as all participants completed identical assessment procedures. Thus, the observed inter-group differences in behavioral performance improvements can still be confidently attributed to the differential impacts of the two game training protocols, rather than uneven practice effects across groups.

The three cognitive tasks used—Distributed Spatial Attention, Spatial N-back, and Color-Direction Conflict—assessed attention, working memory, and executive function, respectively. Accuracy on the Distributed Spatial Attention task increased over time for both the LOL and TKK groups, while reaction time decreased, indicating enhanced attentional function. Accuracy changes for the Spatial N-back task in both groups were modest, with significant improvements observed only for T1 vs. T5 and T1 vs. T6, though reaction times declined across time points. There were no significant differences in accuracy between groups after conducting ANCOVA with the baseline accuracy of the Spatial N-back task (T1) as the covariate. Accuracy on the Color-Direction Conflict task showed only minimal change with no significant differences observed, while reaction times generally decreased. The limited accuracy gains in the Spatial N-back and Color-Direction Conflict tasks may reflect ceiling effects due to initially high baseline performance. Baseline accuracy for these tasks already reached about 80% across all participants at pretest. This high baseline performance left minimal room for further accuracy increments with effective cognitive training. Moreover, when integrating accuracy with response time data—a more sensitive index of cognitive processing efficiency—clear training benefits emerged. After training, response times significantly reduced in the Spatial N-back and the Color-Direction Conflict task with no concomitant decrease in accuracy. These results indicate that training enhanced the speed of cognitive operations without sacrificing response precision, representing an improvement in overall task efficiency that would be overlooked by focusing solely on accuracy metrics. Therefore, significant reductions in reaction times indicate meaningful enhancements in overall task efficiency, working memory, and executive function. These findings suggest that, despite differences in game mechanics, both LOL and TKK impose sufficient cognitive load to facilitate general cognitive improvements. The consistency of these effects across game types suggests a shared underlying mechanism: sustained engagement in high-demand, cognitively challenging, and rewarding environments strengthens cognitive functioning.

While both game types yielded overall cognitive benefits, their impacts were specific to each type. They diverged substantially in both magnitude and long-term retention, driven by the unique core game mechanics of each. The action video game (LOL), which features fast-paced real-time multi-stimulus interactions, continuous peripheral stimulus monitoring, and rapid spatial attention reallocation, yielded superior distributed spatial attention benefits compared to the turn-based strategy game (TKK). That advantage was further evident in the long-term retention of training gains: at the T6 follow-up (vs. T5 post-training), the LOL group maintained larger improvements in Distributed Spatial Attention and Spatial N-back tasks’ performance, while also demonstrating a smaller decline in Color-Direction Conflict task accuracy—findings that signal more robust preservation of cognitive enhancement.

Resting-state EEG reflects spontaneous neural activity during wakeful relaxation, providing a measure of the brain’s intrinsic baseline activity. Relative delta and theta power in resting-state EEG has been linked to neuroplasticity, intrinsic alertness, and neural recovery following cognitive effort [44,45], and enhanced slow-wave activity has been associated with improved cognitive function [46]. Following game training, relative delta and theta power increased significantly in the LOL group, while relative theta power increased in the TKK group. This enhancement may reflect a “baseline shift” in intrinsic brain activity, indicating heightened alertness and readiness to respond efficiently to cognitive demands even at rest. Notably, no significant changes were detected in the relative power of the EEG in other frequency bands (alpha, beta, and gamma). This non-significant result does not imply the absence of potential subtle effects, but rather suggests that the current intervention primarily modulates activity in the delta and theta bands without inducing large-scale reorganization of neural oscillations in other frequency bands. Alpha-band functional connectivity decreased in both groups, with the LOL group exhibiting a greater reduction. This reduction suggests improved neural efficiency, reflecting reduced redundant network communication and a more economical and specialized network architecture [47].

Delta or theta power can reflect diverse physiological states beyond training-related plasticity. To rule out fatigue, arousal, or task-independent factors as potential confounding variables, we implemented the following controls:

First, the EEG data were collected in the eyes-closed resting state, which minimized the influence of arousal levels on the results. Second, the EEG data collection and cognitive testing were scheduled at separate time points, thereby eliminating potential interference of the cognitive task on EEG measurements. Furthermore, strict experimental controls were adopted to mitigate fatigue, including restricting daily training duration and requiring participants to maintain regular sleep schedules.

Changes in resting-state EEG rhythm characteristics are a core noninvasive indicator for assessing neural plasticity, with underlying mechanisms involving synaptic strength remodeling and functional reorganization of neural circuits. In this study, after game training, the relative power of resting-state EEG and brain network connectivity showed specific changes, possibly reflecting enhanced synaptic activity and functional circuit reorganization after training. The enhancement of delta and theta waves is closely related to the synchronization activity of cortical neurons and changes in synaptic connections. At the same time, the decrease in alpha-band brain network connectivity may be consistent with the pruning of irrelevant neural connections, the optimization of network topology, and neural efficiency enhancement. This suggests that game training may drive the structural and functional remodeling of task-related neural pathways. Therefore, current findings suggest that long-term video game training may promote cognitive improvements through the efficient remodeling of resting-state brain function.

Additionally, compared to pre-training (T1), both resting-state EEG measures and behavioral performance on cognitive tasks exhibited the greatest change at the follow-up test, 10 weeks after the completion of training, with the magnitude of change being greater in the LOL group than in the TKK group. This suggests that 30 weeks of video game training could induce robust “offline plasticity”, the consolidation of which persisted beyond the end of active training. Such differences may reflect distinct consolidation timelines associated with the cognitive demands of each game type. LOL emphasizes sensorimotor coordination and rapid decision-making, processes that rely on the gradual optimization of the cortical-striatal system and procedural memory [48], with improvements in neural efficiency (reduced alpha connectivity) and baseline alertness (enhanced low-frequency power) stabilizing over several weeks. In contrast, TKK primarily engages strategic planning and working memory, which depend on hippocampal-prefrontal networks and may be more susceptible to modest attenuation in the absence of continued practice.

The EEG-behavioral correlation analysis clearly revealed the specific correlation patterns between EEG features and cognitive function, providing crucial empirical support for understanding the neurocognitive mechanisms of game training. The relative power of delta and theta waves showed a significant positive correlation with the accuracy of Distributed Spatial Attention and Spatial N-back tasks, and a significant negative correlation with reaction time for both tasks, consistent with the classic functional localization of low-frequency EEG. Notably, the relative power of delta and theta waves did not show a significant correlation with the performance of the Color-Direction Conflict task. This phenomenon may be attributed to the limited improvement in overall cognitive performance after training for this task, resulting in a statistically insignificant correlation between the two EEG indicators. The frontoparietal-occipital functional connectivity of the alpha band network showed a negative correlation with the accuracy of Distributed Spatial Attention and Spatial N-back tasks and a positive correlation with reaction time, but similarly did not establish a significant correlation with performance in the Color-Direction Conflict task.

The core function of the alpha band network is to coordinate the functional division and information transmission of the brain network. The reduction in the alpha band network indicates that training may improve cognitive processing efficiency by simplifying the brain network topology and reducing the redundancy of neural signal transmission. This result also confirms that “training can induce the refinement of the resting-state alpha band brain network.” Overall, these findings suggest that while both types of video game training can enhance cognitive function, the timing, magnitude, and persistence of induced neuroplasticity vary according to the underlying cognitive mechanisms.

## 5. Limitations

Although resting-state EEG provides excellent temporal resolution, its spatial resolution is relatively limited. Future research should integrate task-based paradigms with multimodal neuroimaging techniques (e.g., fMRI or fNIRS) to more comprehensively elucidate the neural mechanisms underlying video game-induced cognitive enhancement. This study had certain limitations in monitoring other game activities of participants. The tracking of participants’ other game activities relied solely on weekly self-reported follow-ups and verification of gameplay duration data from game accounts with voluntary authorization. This approach was prone to missing fragmented gaming behaviors due to subjective biases and suffered from partial data gaps caused by privacy authorization restrictions. It failed to cover non-account-based behaviors such as offline single-player games. Future research could adopt other monitoring methods, such as collaborating with game platforms to obtain de-identified data or having participants supervise each other, thereby enhancing the objectivity and the monitoring process.

Another key limitation of this study is the lack of systematic monitoring of between-group differences in the distribution of academic load during the 10-week post-training phase, relative to participants’ full-year academic schedules. Although we eliminated immediate academic interference by scheduling all assessments outside of exam periods, we did not quantify or balance the variations in academic burden (e.g., intensive coursework, academic competitions) across groups within this post-training window in the context of their annual study plans. This gap prevents full exclusion of potential confounding from uneven academic load distribution across groups, which may have impacted the interpretation of long-term training effect retention. That should be taken into account in future research.

Also, this study lacks cognitive stratification during group allocation, which led to unintended baseline heterogeneity in the Spatial N-back task. We adopted pure randomization prior to baseline cognitive assessments to avoid subjective grouping bias, which increased the variance of behavior data and limited the strict comparability of groups at baseline. Future studies should implement stratified randomization based on baseline performance of key cognitive tasks (e.g., Spatial N-back, Attention Network test) to balance cognitive characteristics across groups from the outset, thereby improving the internal validity of intervention effect conclusions.

Moreover, this study lacks formal measurement and comparison of gaming engagement, enjoyment, and intrinsic motivation between the two training groups. While we standardized training duration and compliance across groups to control for intervention dosage, we cannot rule out the potential influence of differential motivational levels on training outcomes. Future studies should incorporate validated scales (e.g., Game Engagement Questionnaire, Intrinsic Motivation Inventory) to quantify these subjective factors and explore their mediating role in cognitive improvement induced by game-based interventions. Finally, the sample in this study consisted only of young, healthy college students, who may have distinct cognitive capacities, gaming experience, and neural plasticity from other groups. Thus, the generalizability of the findings to children, older adults, or individuals with cognitive impairments remains uncertain. Future research should include participants with more diverse age groups and backgrounds to validate and extend the current findings.

## 6. Conclusions

This 30-week longitudinal study examined the differential effects of training with an action video game (LOL) and a strategy card game (TKK) on cognitive function and resting-state EEG activity. The findings make several key contributions: First, both game types improved attention, working memory, and executive function, highlighting the broad cognitive benefits of interactive digital environments. Second, action game training induced greater increases in low-frequency EEG power and more pronounced reductions in alpha-band functional connectivity, suggesting distinct neural plasticity mechanisms. Furthermore, the persistence of these neural changes in the LOL group at a 10-week follow-up indicates that action gaming promotes long-term consolidation of neural efficiency. These findings suggest that different game mechanics can yield comparable cognitive benefits via distinct neural pathways. Future research incorporating multimodal neuroimaging could further elucidate the neural mechanisms underlying game-induced cognitive enhancement, informing the development of targeted digital interventions tailored to individual neurocognitive profiles.

## Figures and Tables

**Table 1:** Correlation analysis results between behavioral cognitive task indicators and the EEG relative power of the delta band.

Correlation Analysis Results	Accuracy of the Distributed Spatial Attention Task	Reaction Time of the Distributed Spatial Attention Task	Accuracy of the Spatial N-Back Task	Reaction Time of the Spatial N-Back Task	Accuracy of the Color-Direction Conflict Task	Reaction Time of the Color-Direction Conflict Task
*r*	0.3677	−0.3341	0.3326	−0.3160	0.03597	−0.1530
*p*	<0.0001	<0.0001	<0.0001	0.0002	0.6776	0.0754

**Table 2:** Correlation analysis results between behavioral cognitive task indicators and the EEG relative power of the theta band.

Correlation Analysis Results	Accuracy of the Distributed Spatial Attention Task	Reaction Time of the Distributed Spatial Attention Task	Accuracy of the Spatial N-Back Task	Reaction Time of the Spatial N-Back Task	Accuracy of the Color-Direction Conflict Task	Reaction Time of the Color-Direction Conflict Task
*r*	0.3284	−0.2453	0.1999	−0.2788	0.070	−0.1190
*p*	<0.0001	0.0040	0.0197	0.0010	0.416	0.1676

## Data Availability

The data presented in this study are available on request from the corresponding author due to privacy.

## Abbreviations

The following abbreviations are used in this manuscript:

EEG	Electroencephalography
LOL	League of Legends
TKK	Legends of the Three Kingdoms
ANOVA	Analysis of Variance
ANCOVA	Analysis of Covariance
PLV	Phase-Locking Values
FFT	Fast Fourier Transform
